# Maintaining non-communicable disease (NCD) services during the COVID-19 pandemic: lessons from Thailand

**DOI:** 10.1136/bmjgh-2023-014695

**Published:** 2024-10-22

**Authors:** Melanie Coates, Paul Li Jen Cheh, Thanathip Suenghataiphorn, Wasin Laohavinij, Aungsumalee Pholpark, Natchaya Ritthisirikul, Sirithorn Khositchaiwat, Piya Hanvoravongchai

**Affiliations:** 1 Faculty of Medicine, Chulalongkorn University, Bangkok, Thailand; 2 Global Health Partnerships, Health Education England, London, UK; 3 National Health Foundation, Bangkok, Thailand

**Keywords:** COVID-19, Global Health, Health services research, Health systems, Public Health

## Abstract

The COVID-19 pandemic presented a significant challenge to health systems worldwide, requiring resources to be directed to the pandemic response while also maintaining essential health services. Those with non-communicable diseases (NCDs) are particularly vulnerable to COVID-19, and interrupted care resulting from the pandemic has the potential to worsen morbidity and mortality.

We used narrative literature review and key informant interviews between August 2021 and June 2022 to identify how NCD services were impacted during the pandemic and which good practices helped support uninterrupted care.

On the background of an existing strong healthcare system, Thailand exhibited strong central coordination of the response, minimised funding interruptions and leveraged existing infrastructure to make efficient use of limited resources, such as through mobilising healthcare workforce. A key intervention has been redesigning NCD systems such as through the ‘New Normal Medical Services’ initiative. This has promoted digital innovations, including remote self-monitoring, patient risk stratification and alternative medication dispensing. Emphasis has been placed on multidisciplinary, patient-centred and community-centred care.

NCD service utilisation has been disrupted during the COVID-19 pandemic; however, newly adapted efforts on top of existing robust systems have been critical to mitigating disruptions. Yet challenges remain, including ensuring ongoing evaluation, adaptation and sustainability of redesign initiatives. This learning offers the potential to further positive health systems change on a wider scale, through sharing knowledge, international collaboration and further refinement of the ‘new normal’ model.

**Summary Box**

- During the COVID-19 pandemic, healthcare systems faced significant pressures to provide COVID-19 care while maintaining essential service provision, such as for non-communicable diseases (NCDs).
- Those with NCDs require regular and repeated health interactions, therefore, there is substantial value in understanding how we can maintain these services while balancing the competing demands on healthcare during a pandemic.
- We identified good and innovative practices demonstrated by Thailand in maintaining NCD services during the pandemic, such as the ‘New Normal Medical Services’ initiative.
- Learning from Thailand’s experiences has the potential to benefit countries in strengthening services and cultivating more resilient systems in the face of future pandemics.

## Introduction

 The COVID-19 pandemic has challenged health services and systems resilience worldwide. Countries have faced unprecedented pressures on resources for pandemic response, alongside maintaining essential health services (EHS).

During a pandemic, reduced access to EHS, such as for non-communicable diseases (NCDs), is known to worsen health outcomes and compound the direct effects of the pandemic on morbidity and mortality.^1^ The Ministry of Public Health (MOPH) has named NCDs as the number one health issue facing Thailand.^2^ NCDs are a major cause of morbidity and mortality, accounting for over 75% of all deaths in Thailand in 2019.^3^ Through both direct and indirect costs, NCDs cost the Thai economy 1.6 trillion THB each year, corresponding to 9.7% of the country’s gross domestic product in 2019.^4 5^

In Thailand, primary healthcare (PHC) is the main provider of NCD services. NCD care accounts for a major proportion of its workload in providing disease prevention, monitoring, treatment, rehabilitation and health promotion services.^6^ PHC is primarily delivered via Tambon (subdistrict) Health Promotion Hospitals. These outpatient centres are mostly staffed by nurse practitioners and public health officers, though some large centres have physicians. PHC centres are supported by district hospitals that provide medicines and take referrals of patients with poorly controlled diseases.^6^

COVID-19 has impacted the provision and demand for NCD services.^7^ In 2021, a survey of approximately 600 public medical facilities throughout Thailand found that 51% experienced disruptions in diabetes and hypertension clinics.^8^ Those with NCDs are at increased risk of becoming severely ill from COVID-19, with vulnerable groups, such as those living in poverty, even more at risk.^9 10^ As the chronic nature of NCDs requires regular and repeated healthcare interactions, interrupted care may cause increased disability, premature death and wider economic effects. Therefore, it is vital to understand the effects of the pandemic on NCD care and how to mitigate service disruptions.^11^

This manuscript aims to identify how NCD services were impacted during the pandemic and which good practices helped support service maintenance. Data collection occurred between August 2021 and June 2022 through a narrative literature review and key informant (KI) interviews. Literature was selected through searching electronic databases, grey literature and sources identified by KIs. Literature included published and unpublished articles, policy announcements, and other documents, published in both Thai and English languages. 20 KIs were interviewed either via telephone or Zoom calls between August 2021 and April 2022. The sample was representative of personnel from a broad spectrum of areas in health systems research, policy-making bodies, private and public hospitals, PHC centres, civil society, and non-governmental organisation representatives (see online supplemental appendix A).

### NCD service maintenance during COVID-19 in Thailand

#### Existing strong health systems

Thailand is well known for its strong health system, with decades of investment in health infrastructures and workforce capacity.^12^ The government’s long-standing commitment to developing health infrastructure in Thailand has resulted in a robust and well-resourced system. This afforded Thailand a responsive and flexible system that could adapt to changing demands during the pandemic.^13^ In particular, KIs cited universal health coverage, successfully implemented in 2002, as a cornerstone of the existing strong health system, improving access to healthcare by increasing coverage for citizens and increasing numbers of health centres in rural areas.^14^

#### Centrally coordinated response

The NCD response has been centrally led by the MOPH during the COVID pandemic, including through releasing policy guidelines, research and national redesign efforts for NCD services, titled the New Normal Medical Services.^13^ Centralised initiatives enable a more cohesive and efficient national response, with potential for rapid budget mobilisation, sustained funding, more effective engagement of necessary bodies, coordination with other public and civil society institutes and redeployment and upskilling of human resources.^15^ Common guidelines standardise care, improving quality and accessibility, and potentially reducing inequalities.^16^

The budget for secondary prevention of NCDs was increased from 270 million baht in 2010–1.06 billion baht in 2019, representing government prioritisation of NCDs.^4^ The MOPH has established distinct teams for NCDs, for example, the Thai Healthy Lifestyle Strategic Management Office, the Bureau of Noncommunicable Diseases and the Bureau of Tobacco Control. This ensures earmarked funding and NCD-specific work by these groups, allowing greater expertise to develop in these areas.

#### Minimal funding disruptions

During 2020, there were minimal funding disruptions of routine public health services such as NCDs.^13 17^ Disruptions to EHS financing were minimised by adopting a prepayment system with capitation payments for outpatient services, ensuring budgets were not impacted by COVID-19. In addition, COVID-19 services were funded separately from other health services.^17^ The government mobilised extra funds, mainly from government central budget, to fund the COVID-19 response or used existing funds, such as the social security fund which was used to provide COVID-19 treatment to their beneficiaries.^17 18^ Further funding was mobilised from the existing community health fund (CHF), which provides support at the community level. The fund is subsidised by the National Health Security Office (NHSO) and devolves power to local administrations.^19^ It was previously underused; however, uptake increased during the pandemic, attributed to NHSO issuing notifications to encourage use of CHF and accelerating processes for project approval during the pandemic. This allowed local administrative organisations to directly approve health-related projects costing less than THB100 000 per project during the pandemic.^20^,^22^

A KI from an urban PHC centre in the north of Thailand reported that NCD service redesign during the pandemic, such as telephone costs and case managers, was able to be delivered using existing resources. However, a KI from the Department of Medical Services (DMS) reported that there is a need for more earmarked funding for EHS.

#### Optimisation of healthcare workforce

There was adequate health workforce capacity to respond to the pandemic, which minimised the impact on EHS such as NCDs.^23^ Prior to the pandemic, the government more than doubled the number of qualified nurses and midwives and almost tripled the number of doctors between 2002 and 2018, achieving 8.97 doctors and 30.8 nurses and midwives per 10 000 population by 2019.^24^ Mandatory rural service and financial and non-financial incentives were implemented to improve the rural distribution of healthcare workers.^25^ Village health volunteers (VHVs), health coaches and case managers support the PHC system and NCD care at the community level and provide skilled care while reducing reliance on nurse and physician staff. During the pandemic, the Department of Disease Control (DDC) advised hospitals to designate separate health workforces for COVID-19, ensuring protected staffing for EHS.^26^ Thailand also has robust civil society and community-based networks, which support those with chronic diseases, particularly in vulnerable populations and during the pandemic.^27^ These networks were activated during the pandemic, enabling a strong multisector response with greater collaboration between public and private sector.^1327^,^29^

However, there are still strides to be made in developing the healthcare workforce. A KI from the DMS reported that work is needed to develop responsive systems for more dynamic workforce planning. Workforce density remains below the UN Sustainable Development Goals threshold of 44.5 physicians, nurses and midwives per 10 000 population.^30^

#### VHV network

A national programme of over 1 million VHVs support healthcare delivery at the community level. KI interviews of VHVs and representatives of the Society of VHVs spoke to the critical role that VHVs played in the pandemic, particularly in NCD care. KIs reported VHVs have diverse roles in supporting NCD care such as through health education and promotion, diabetes and hypertension screening in target populations, home visits, medication delivery, and identifying and referring patients to secondary care when needed.^12^ In a survey of 589 healthcare facilities in July 2020, 73% reported using VHVs to deliver medicines during the pandemic, with 77% using VHVs to follow-up NCD patients, and 64% for health knowledge promotion for NCDs.^8 31^

### PHC service redesign

#### The New Normal Medical Services initiative

In 2020, the DMS, MOPH, established the national New Normal Medical Services initiative across the health system. They released comprehensive guidelines and checklists for 13 EHS hospital departments, including NCDs.^32^ This aimed for patients to receive appropriate treatment and care (for both COVID-19 and non-COVID-19), and to prepare the system for future health emergencies. The model specifically focused on three outcomes: non-crowding to reduce risk of virus spread, patient and health personnel (2P) safety and health equity. The model was initially piloted in Pattani province, with learning used to expand guidance for other provinces under the New Normal Medical Services initiative.

**Figure 1:**
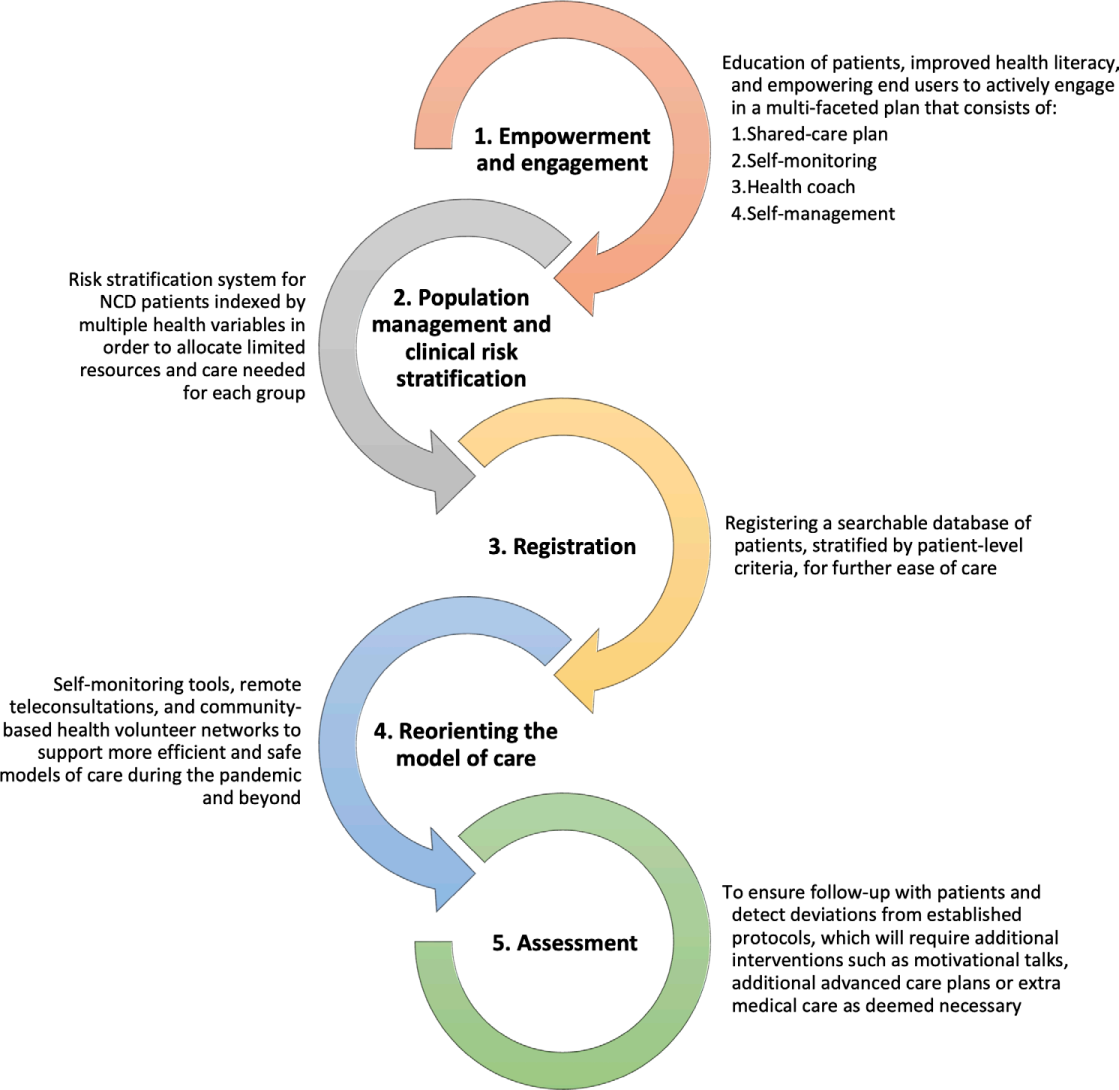
Five-step plan for New Normal Diabetic and Hypertensive Clinic Redesign. Adapted from the Handbook of Integrated, People-Centred Health Services in New Normal Diabetic and Hypertensive Clinic, MOPH. MOPH, Ministry of Public Health; NCDs, non-communicable diseases.

As part of the initiative, the *Handbook of Integrated, People-Centred Health Services in New Normal Diabetic and Hypertensive Clinic* was released by the DMS in 2020. This formalised guidelines for diabetes and hypertension services nationally and established a five-step plan, informed by the WHO’s Framework for Integrated People-Centred Health Services and value-based healthcare (see figure 1).

Shared care plans create patient-centred programmes with a focus on multidisciplinary collaboration. Responsibility for patient care is shared across secondary and tertiary hospitals, community services and the patients themselves. Patients work with providers to identify personalised health goals and develop treatment plans in line with their conditions and life circumstances.

Patients are empowered to self-monitor, for example, blood pressure or blood sugar levels, supported by health coaches (community health workers or VHVs), who provide knowledge, advice and motivational skills. Self-management aims for patients to understand their disease severity, how to manage their treatment and symptoms, and when to seek help appropriately.

Patients are stratified according to clinical risk (see table 1). For patients requiring in-person health services, redesigned pathways reduce risk of COVID-19 exposure and transmission, for example, by observing physical distancing and improving ventilation. Patients can receive medicines through several channels including community drugstores, drive-through systems at health centres and hospitals, postal service or delivery to their home.

**Table 1:** Clinical risk stratification groups

Good control	Low-risk group with mild to no complications	Patients receive advice via telemedicine and are permitted to visit their doctor in person every other recurring appointment (approximately every 4 months)
Good-moderate control	Moderate-risk group with moderate complications	Patients are referred for in-person and/or telemedicine-based care with varying frequency based on severity of condition (approximately every 3 months in person)
Poor-moderate and poor control	High-risk group with severe complications	Patients are referred for in-person care for every visit, whereby care from doctors and a multidisciplinary team is made available and includes follow-up home visits by public health volunteers

KIs from a PHC centre in the north of Thailand reported that the centre was very crowded prior to the pandemic, with 3 physicians seeing approximately 100 patients per day (around 2–3 min per patient). In 2018, they implemented a traffic light system, stratifying patients with diabetes or hypertension into green, yellow or red groups, with individualised care plans and adjusted monitoring frequency based on patient need.

A KI from a public hospital in the Central Region reported that it was easier to implement redesign in primary care during the pandemic than before. The centre also introduced patient stratification, with self-monitoring for green groups, sharing results with providers via the LINE messaging application. For red-group patients, this enabled more one-to-one consultations and in-depth health education, supporting development of personalised care plans and shared decision-making between patients and health professionals. Medications were delivered to patients or collected from community pharmacies, reducing crowding.

In Pakkred district, adaptations included a fast-track system for vulnerable patients (eg, elderly and high-risk groups), and an appointment system to reduce crowding. Screening, measurements, consultation and medicine dispensing were arranged in one place to reduce movement within hospitals. These combined interventions resulted in shorter visit times, reportedly from 2 to 3 hours to 1 hour.^33^

A KI from a district public hospital in the Central Region reported that following NCD redesign between 2021 and 2022, patients with good diabetic control increased from 30% to 56% and patients with well-controlled hypertension rose from 50% to 73%. Outpatient visits for diabetes and hypertension were reduced from 160 to 70 patients per day. An urban PHC centre in the North reported that 53% of patients had better control of their blood sugar following system redesign with health coaches after 6 months. Both KIs reported high patient satisfaction rates with redesigned services.

According to the Health Data Centre, diabetes and blood pressure screening among Thais aged over 35 remained stable throughout the pandemic at around 90% (see online supplemental appendix B). Blood sugar and blood pressure control saw a slight improvement between 2019 and 2021. However, eye and foot screening for diabetes was more negatively impacted during this time, possibly due to the requirement for in-person physical examinations. Chronic kidney disease screening, however, saw only a minor decrease in screening rates. This may indicate that laboratory testing was sustained more than in-person services during the pandemic.

#### Digital health services

Mobile phone applications have been developed for NCD prevention and service delivery, including self-checks, remote appointment booking and health education.^34^ KIs in the North region reported that keeping paper records was challenging as records could be lost by patients, for example, when recording blood sugar and blood pressure. Therefore, the centre collaborated with a university to develop an application for real-time recording at check-in-points. Another NCD self-monitoring app, Mor Rujak Khun (‘Doctor Knows You’), was developed between Naresuan University and the MOPH and implemented in the Pattani pilot, to support real-time self-monitoring, with data analysed by medical staff at the district or subdistrict level.

Use of digital health technologies has been widely adopted by patients and staff, as many interventions use existing systems, such as calls and texting over the phone, and LINE or other popular social media apps. KIs in PHC and community settings reported using LINE messaging application to communicate with patients and between healthcare staff. In Pakkred, LINE was also used, with online consultation made available 24/7 through these accounts. A Facebook page was set up for inquiries from patients and the telemedicine ‘PK COVID’ programme provided teleconsultation over the phone with doctors.^33^

Universities and private companies have also developed medical robots, for example, Pinto Robot by Chulalongkorn University, to support food and medicines delivery, remote monitoring and communication between medical teams and patients, minimising exposure to infection.^35^

The NHSO introduced additional incentives to providers for using telemedicine during the pandemic, with an additional 30 baht per visit on top of the standard reimbursement rate. Between April and September 2021, NCD-specific telemedicine reimbursements from the NHSO increased from 3767 to 73 338 visits and increased coverage from 14 to 35 hospitals across the country.^36^

Thai homes have internet access at a rate above the global average, with increasing numbers of Thai citizens using the internet each year (78% in 2020 from 67% in 2019), therefore, digital services are likely to be received well by the public.^37 38^ However, the number of households with access to computers in Thailand is below the global average (21% vs 49%), with many solely using mobile devices, therefore, new technologies will need to take this into consideration.^38^ For at-risk groups or in rural areas, digital health may permit specialist care not usually available, mitigate indirect costs such as travel and time off work and reduce the risk for those who face discrimination in clinical spaces, such as migrant groups or LGBTQIA+ (lesbian, gay, bisexual, transgender, queer, intersex, asexual) populations.

## Conclusion

Thailand has exhibited innovative practices in maintaining NCD services and accelerating positive health systems transformation during the pandemic. Long-standing investment has resulted in resilient and adaptable services. Leveraging existing assets has provided cost-effective adaptations which can be implemented quickly in the face if competing demands from COVID-19. There have been minimal funding disruptions to EHS, which has supported service maintenance and innovation during the pandemic.

There is a wide potential reach of NCD redesign, with standardised guidelines allowing a coordinated and cohesive system countrywide. Through redesign, the patient pathway has been restructured for more efficient flow. Clinical risk stratification allocates limited resources while prioritising clinical safety, and patient-centred care may improve patient satisfaction and increase adherence to care plans.^39 40^ Services devolved to the community through self-monitoring, check-in points, health coaches and VHV support. PHC is considered one of the most effective strategies in the prevention and management of NCDs, therefore, continuing to strengthen PHC will help cultivate sustainable systems going forward.^41 42^

Patients should be engaged in decision-making to ensure that services meet community needs. Health equity should be prioritised to protect vulnerable groups such as the elderly, migrant workers, refugees and those of low socioeconomic status. Existing community engagement methods including VHVs, civil society and patient advocacy groups may be key links to target communities.^7^ Digital health innovations increase access to services and will be key in preparing for future health emergencies. Thailand has relatively high rates of internet usage, offering a good foundation for the widespread adoption of digital interventions.

NCD prevention should be prioritised, such as through investment in PHC for early screening and risk factor management. Lessons can be learnt from other countries, such as Bhutan, which introduced their service with care and compassion initiative, in which all persons visiting health facilities are screened for NCDs. Similarly to Thailand, this initiative introduces a people-centred approach at the district level. Key components include home care, improved access to medicines, mentoring of PHC workers and real-time monitoring.^43^ These initiatives may also be of benefit in Thailand, however, different contexts and systems will need to be considered in translating learning. Sharing experiences and learning from NCD service implementation, in particular during the pandemic, on regional and global levels will benefit countries in improving their own services and cultivating more resilient systems in the face of future pandemics.

In all, NCD service redesign practices are likely to be sustained beyond the current pandemic, however, ongoing monitoring and adaptation of the redesign will be key in ensuring effectiveness and equitable access and outcomes for patients. Evaluation and refinement of the model will be necessary to prepare for future potential health emergencies. High-quality and accessible NCD monitoring systems are needed to facilitate learning and quality improvement, and long-term financing methods should be considered to ensure sustainability of the initiative.

## Supplementary material

10.1136/bmjgh-2023-014695 online supplemental file 1

10.1136/bmjgh-2023-014695 online supplemental file 2

## Data Availability

Data are available on reasonable request.
