# Supplementary material for: Maintaining non-communicable disease (NCD) services during the COVID-19 pandemic: lessons from Thailand
Source: BMJ Glob Health. 2024 Oct 22;8(Suppl 6):e014695. doi: 10.1136/bmjgh-2023-014695 (PMC11733076; doi:10.1136/bmjgh-2023-014695)
Supplement: online supplemental file 2 [file bmjgh-8-Suppl_6-s002.pdf]

Appendix A – Methods

Data collection occurred between August 2021 and June 2022 through narrative literature review and key informant (KI) interviews. Literature was selected by searching electronic databases, grey literature sources, and sources identified by KIs. Electronic databases searched were PubMed, Google Scholar, the Official Website of Ministry of Health, and international organization websites, including the WHO, UN, OECD, Word Bank, and news websites. Key terms included COVID-19, essential health services, NCD, health services. Literature included published and unpublished articles, government policy documents and announcements, and other documents, published in both Thai and English languages. 114 articles/documents were included. Ethical approval was obtained from Chulalongkorn University Institutional Review Board. All demographic data and quotes were anonymised.

20 KIs were recruited for semi-structured interviews in a two-stage process using purposive and snowball sampling. 11 participants were interviewed in the first round to acquire a high-level overview of the policy and health systems response to COVID-19 and to identify good practices for maintaining EHS in Thailand. The research team selected good practices in EHS maintenance based on the following criteria: mentioned by multiple informants; initial quantitative and/or qualitative outcomes available; an innovative, unique, adaptive or responsive practice; potential for impact; potential to scale; overall reach and access; and equity considerations. Following first-round interviews and initial literature review, maintaining NCD service delivery, in particular primary care for hypertension and diabetes, was identified as a good practice to be investigated in-depth. In the second round, 9 additional participants were recruited to focus on maintenance of NCD services during the pandemic. In total, 22 KIs were approached with 2 declining to participate.

Interviews were conducted either via telephone or Zoom calls between August 2021 and April 2022. Semi-structured in-depth interview guides were developed in English and translated into Thai for data collection. Interviews were audio-recorded, and field notes were collected. 19 interviews were conducted in Thai language and 1 in English. The sample was representative of personnel from a broad spectrum of areas in health systems research, policy-making bodies, private and public hospitals, primary health care centres, civil society and non-governmental organisation representatives (see Table 1). Patients or the public were not involved in the development, design, recruitment to or conduct of this study.

Table 1. Key Informant Affiliations

Organisation type	Affiliation
**Regional**	Tertiary public hospital in Northeast region
	Private hospital group
	District public hospital in Central region
	University hospital
	PHC centre, Nakhon Si Thammarat
	Provincial hospital in North region
	District public hospital in Central region
	Urban PHC centre under provincial hospital in North region
	Village Health Volunteer, Sukhothai province
**Central**	Department of Medical Sciences (DMS)
	Department of Medical Services
	Department of Disease Control (DDC)
	Department of Disease Control (DDC)
	Health Systems Research Institute (HSRI)
	Society of Village Health Volunteers in Thailand
	Division of Non-Communicable Diseases, Department of Disease Control (DDC)
	Information and Communication Technology (ICT) Center, Office of the Permanent Secretary of Ministry of Public Health
	Field Epidemiology Training Programme (FETP)
**Other**	AIDS Access Foundation
	WHO Thailand

Appendix B - Table 2. NCD service disruption during the pandemic

	Fiscal Year (October - September)				
**Indicators**	**2017**	**2018**	**2019**	**2020**	**2021**
**General Population Screening**					
**% of population aged 35 years and above who received diabetes screening**	85.9	88.4	89.4	90.7	89.9
**% of population aged 35 years and above who received blood pressure screening**	88.0	88.9	89.7	90.9	90.1
**Diabetes and hypertension related outcomes**					
**% of diabetes patients who had controlled blood sugar**	23.3	27.1	28.4	29.6	29.3
**% of hypertensive patients who had controlled blood pressure**	37.5	42.6	44.6	44.3	47.9
**Diabetes and hypertension related quality of care indicators**					
**Proportion of diabetes patients that received annual eye screening**	53.2	56.2	57.2	53.7	46.5
**Proportion of diabetes patients that received annual foot screening**	59.0	64.2	62.7	58.5	52.9
**Proportion of diabetes and/or hypertensive patients that received annual chronic kidney disease screening**	48.3	63.2	64.0	63.5	62.7
